# Renal Proteome in Mice with Different Susceptibilities to Fluorosis

**DOI:** 10.1371/journal.pone.0053261

**Published:** 2013-01-04

**Authors:** Juliane Guimarães Carvalho, Aline de Lima Leite, Camila Peres-Buzalaf, Fernanda Salvato, Carlos Alberto Labate, Eric T. Everett, Gary Milton Whitford, Marília Afonso Rabelo Buzalaf

**Affiliations:** 1 Department of Biological Sciences, Bauru Dental School, University of São Paulo, Bauru, São Paulo, Brazil; 2 Department of Genetics, Escola Superior de Agricultura “Luiz de Queiros”, University of São Paulo, Piracicaba, São Paulo, Brazil; 3 Department of Pediatric Dentistry, School of Dentistry, The Carolina Center for Genome Sciences, University of North Carolina at Chapel Hill, Chapel Hill, North Carolina, United States of America; 4 Department of Oral Biology, School of Dentistry, The Medical College of Georgia, Augusta, Georgia, United States of America; UGent/VIB, Belgium

## Abstract

A/J and 129P3/J mouse strains have different susceptibilities to dental fluorosis due to their genetic backgrounds. They also differ with respect to several features of fluoride (F) metabolism and metabolic handling of water. This study was done to determine whether differences in F metabolism could be explained by diversities in the profile of protein expression in kidneys. Weanling, male A/J mice (susceptible to dental fluorosis, n = 18) and 129P3/J mice (resistant, n = 18) were housed in pairs and assigned to three groups given low-F food and drinking water containing 0, 10 or 50 ppm [F] for 7 weeks. Renal proteome profiles were examined using 2D-PAGE and LC-MS/MS. Quantitative intensity analysis detected between A/J and 129P3/J strains 122, 126 and 134 spots differentially expressed in the groups receiving 0, 10 and 50 ppmF, respectively. From these, 25, 30 and 32, respectively, were successfully identified. Most of the proteins were related to metabolic and cellular processes, followed by response to stimuli, development and regulation of cellular processes. In F-treated groups, PDZK-1, a protein involved in the regulation of renal tubular reabsorption capacity was down-modulated in the kidney of 129P3/J mice. A/J and 129P3/J mice exhibited 11 and 3 exclusive proteins, respectively, regardless of F exposure. In conclusion, proteomic analysis was able to identify proteins potentially involved in metabolic handling of F and water that are differentially expressed or even not expressed in the strains evaluated. This can contribute to understanding the molecular mechanisms underlying genetic susceptibility to dental fluorosis, by indicating key-proteins that should be better addressed in future studies.

## Introduction

The widespread use of F has contributed to the caries decline, but excessive intake may affect both bone metabolism and enamel development, causing skeletal and dental fluorosis, respectively. There are many sources of F intake, such as drinking water, dental products, dietary supplements and infant formulas [1]. There is evidence that the prevalence of dental fluorosis (DF) is increasing worldwide both in fluoridated and non-fluoridated communities [2]. In the US, 23% of 6- to 39-yr-old subjects present enamel fluorosis, ranging from very low to relatively high in severity [3]. However, the exact mechanisms by which F affects biomineralization are not completely understood [4], [5]. It has been proposed that genetic determinants influence the susceptibility to DF in humans [6] and mice [7]. Two strains of mice have been identified with distinct responses to the effects of F in the mineralized tissues. The A/J strain is “susceptible”, with a rapid onset and severe development of DF, while the 129P3/J is “resistant”, with minimum development of DF [7]. These strains also differ regarding their susceptibilities to the effects of F in bone [8], [9].

To determine whether such differences were due to differences in F metabolism, we conducted a metabolic study in which total F intake and excretion were measured. Our results showed that, compared to A/J mice, 129P3/J mice ingested less water, excreted less urine, had lower urinary F excretion and consequently had higher F retention and plasma and femur F levels [10]. However, these findings were not able to explain the mechanisms underlying the differences in the metabolic handling of F.

Kidneys represent the major route of removal of F from the body [11]. After F enters the renal tubules, a variable amount is reabsorbed, depending on the urinary pH because transmembrane migration occurs by diffusion of HF [12]. Thus, any factor that affects urinary pH will have an impact on the amount of F that is excreted in urine [11]. Urinary F excretion is also influenced by glomerular filtration rate since its reduction, as occurs in chronic renal dysfunction as well as in the last decades of life, results in lower excretion and increased plasma F levels [13]. Considering that kidney is a key organ in the metabolism of F, we then sought to investigate the molecular mechanisms underlying the renal F metabolism in A/J and 129P3/J mice that may account for their differential metabolic handling of F. To address this, proteomic analyses were performed on kidneys of A/J and 129P3/J mice receiving both low and high level of F-containing water.

## Materials and Methods

### Animals and Treatment

Male mice from the A/J and 129P3/J inbred strains (3-week-old) were randomly distributed into three groups (n = 6/strain) based on the F concentrations in the drinking water. All animals were housed in pairs in metabolic cages with *ad libitum* access to low-F food (AIN76A, PMI Nutrition, Richmond, IN, USA, 0.95 mg/Kg F) and water, to allow analysis of water and food consumption [10]. The temperature and humidity in the climate-controlled room, which had a 12-h light/dark cycle, were 23±1°C and 40%–80%, respectively. All experimental protocols were approved by the Ethics Committee for Animal Experiments of Bauru Dental School, University of Sao Paulo (Protocol # 026/2007). Experimental groups received drinking water containing 10 (low) or 50 (high) ppm F ion (as NaF), for 60 days. Control group received deionized water for the same period. At the end of the study, the mice were anesthetized with ketamine/xylazine and kidneys were collected. The left kidney was washed with cold buffer containing Tris 100 mM, EDTA 1 mM, PMSF 1 mM, pH 7.4, frozen at liquid nitrogen and kept at -80°C until proteomic analysis. The right kidney was collected for F analysis.

### F analysis in kidney

Kidneys were homogenized in deionized water for 2 min using a homogenizer (Marconi, Model MA 102). Kidney F concentrations were determined in duplicate (100 mg of kidney tissue) after overnight hexamethyldisiloxane (HMDS)-facilitated diffusion [14], [15] using the ion-specific electrode (Orion Research, Model 9409) and a miniature calomel electrode (Accumet, #13-620-79) both coupled to a potentiometer (Orion Research, Model EA 940). F standards (0.00475 to 0.19 µgF) were prepared in triplicate and diffused in the same manner as the samples. In addition, nondiffused standards were prepared to have exactly the same F concentrations as the diffused standards. Comparison of the mV readings demonstrated that the F in the diffused standards had been completely trapped and analyzed (recovery>95%). The mV potentials were converted to µg F using a standard curve with a coefficient correlation of r≥0.99.

### Sample Preparation for 2DE

Kidney samples were homogenized using mortar and pestle in liquid nitrogen. Denaturation buffer (7 M urea, 2 M de thioureia, 4% CHAPS, 1% DTT and 0.5% IPG pH 3–10, GE Healthcare, Uppsala, Sweden) was added. After 1 h vortexing at 4°C, samples were centrifuged at 25000×*g* for 30 min at 4°C for supernatants collection. The proteins were precipitated by using the kit *PlusOne 2D Cleanup* (GE Healthcare, Uppsala, Sweden), as recommended by the manufacturer. The pellets were resuspended in rehydration buffer (8 M urea, 0.5% CHAPS, 10% glycerol, 0.5% IPG buffer pH 3–10, 7 mg/2.5 mL DTT, 0.002% bromophenol blue). Protein concentration was measured in each sample by Bradford protein assay. After quantification, 1000 µg of kidney proteins from each animal of the same group and strain were combined to constitute a pool [16] that was submitted to proteomic analysis in triplicate, as described below.

### 2-DE Separation

Renal proteins (1000 µg) were taken from each pooled sample and mixed in rehydration buffer to a volume of 400 µL which was then loaded onto 24-cm IPG strips (linear pH 3–10). Rehydration and first-dimensional IEF were performed on IPGphor IEF system at 20°C with the following parameters: 50V for 12 h, 500V for 1 h, 1000V gradient for 1 h, then 10000 V for a total 40,000 V. *Ettan DALTsix* (GE Healthcare) (GE Healthcare, Uppsala, Sweden) with homemade12.5% acrylamide gels was used for the second dimension separation. Electrophoresis was performed at 15 mA/gel (80V) for 1 h and at 60 mA/gel (500V) until bromophenol blue line had reached the bottom of the gels. The resolved protein spots were stained with Colloidal Coomassie Brilliant Blue G-250 [17]. Gels were scanned with an Imagemaster scanner, and all images were analyzed by ImageMASTER 2D Platinum 7.0 software (GE Healthcare, Uppsala, Sweden). Parameters used for spot detection were minimal area = 5 pixels; smooth factor = 4; and saliency = 100. The gel chosen as the reference had the highest number of spots. The reference gel was then used for matching of corresponding protein spots between gels. Following average mode of background subtraction, individual spot intensity volume was normalized with total intensity volume (summation of the intensity volumes obtained from all spots in the same 2-DE gel). The normalized intensity volume values of individual protein spots were then used to determine differential protein expression between control and experimental groups. 2D spots that exhibited a twofold or more decrease or increase were tested for statistical significance. Analysis of 2D-gel variability among the replicates of each experimental condition was taken by using the relative volume (% vol). The correlation coefficients among the triplicates are shown to vary from 0.9385 to 0.9821 (Figure S4 - Supplementary information).

### LC-MS/MS Analysis

Protein spots of interest were excised from the gel and destained three times with 25 mM Ambic/Acetonitrile (50∶50 v/v) for 30 min. The destained gel pieces were dehydrated twice with acetonitrile (ACN) for 10 min and dried in a vacuum concentrator (Eppendorf, Hamburg, Germany). The dried gel pieces were rehydrated with 20 mM DTT in 50 mM Ambic for 40 min at 56°C. Excess of reagent was removed and 55 mM iodoacetamide (IAA) in Ambic 50 mM was added for 30 min RT at dark. The remaining liquid was removed and washed out with 25 mM Ambic, followed by dehydration with ACN. After its removal, the gels were dried again. For digestion, dried gels were incubated with 10 ng/µL trypsin in 25 mM Ambic for 15 min (Trypsin Gold Mass spectrometry, Promega, Madison, USA). Peptides were sequentially extracted from the gels initially in 50% ACN (v/v) with 5% formic acid for 14 h at 37°C, then in 50% ACN (v/v) with 1% formic acid for 15 min, followed by 60% methanol (v/v) with 1% formic acid for 15 min and twice with 100% ACN at 45°C under sonication (40kHz/30W, Branson, Danbury, USA). Extracts were dried using a vacuum concentrator (Eppendorf, Hamburg, Germany) and kept at -20°C. Prior to MS identification, dried peptides were dissolved in 12 µL 0.1% formic acid. The peptides were identified and quantified by LC-ESI-Q-TOF MS (Liquid Chromatography Electrospray Ionization Quadrupole Time of Flight Mass Spectrometry) (Waters, Mildord, USA). MassLynx 4.1 SCN662 software (Waters, Mildord, USA) was used to submit the combined MS and MS/MS data to MASCOT database search engine (http://www.matrixscience.com) (version 2007.12.04) based on IPI (International Protein Index) protein database restricted to taxonomies *Mus musculus* (Mouse). The search was limited with a mass tolerance of 100 ppm and only one missed cleavage per peptide was allowed. For modification of peptides, cysteine carbamido-methylation (fixed) and methionine oxidation (variable) were considered. Significant matching protein required score of *>*60. Accuracy between the theoretical and experimental obtained mass and pI were also considered. When 2 or more proteins with high scores were identified in the same spot, they are excluded from analysis. Identified proteins were classified into 6 different categories according to their primary function [18].

### Statistical Analysis

For kidney F concentration, the software GraphPad InStat version 4.0 for Windows (GraphPad software Inc., La Jolla, USA) was used. Data were analysed by 2-way ANOVA and Bonferroni test for individual comparisons (p<0.05).

For proteomic data, statistical analysis was performed using ANOVA available at ImageMaster 2D Platinum 7.0 software (GE Healthcare, Uppsala, Sweden). Only proteins with significantly altered levels were excised for identification by LC-MS/MS (p<0.05).

## Results

### Renal F Concentration

Mean kidney F (±se) concentrations for A/J mice for control, 10 ppmF and 50 ppmF groups were: 0.126±0.008, 0.174±0.007 and 0.296±0.026 µg/g. The corresponding values for 129P3/J mice were 0.139±0.015, 0.163±0.010 and 0.198±0.046 µg/g, respectively. Two-way ANOVA revealed a significant difference among the treatments (F = 35.13, p<0.0001), but not between the strains (F = 0.099, p = 0.756) without significant interaction between these criteria (F = 0.124, p = 0.884). For both strains, significantly higher kidney F concentrations were found for the 50 ppmF group, when compared with control and 10 ppmF groups that did not significantly differ from each other.

### Identification of Differentially Expressed Proteins

**Figure 1:**
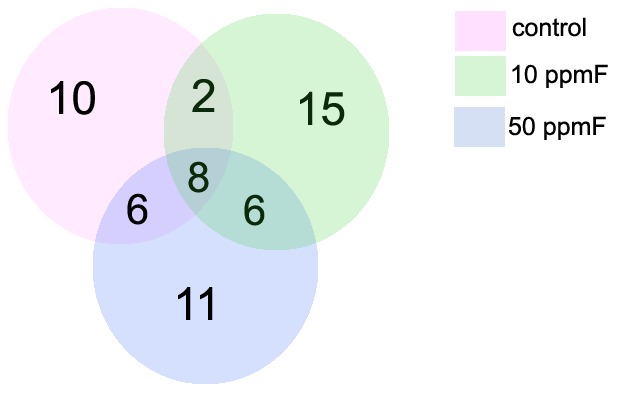
Venn diagram showing distribution of total kidney proteins identified with differences in expression from the 2D-PAGE and LC-MS/MS-based proteome. The numbers indicate the total protein identified from each comparison (control, 10 and 50 ppmF A/J and 129P3/J) and the number of proteins commonly identified between them.

For the statistical analysis, comparisons were performed between the strains as follows: Control groups (A/J *vs* 129P3/J mice), 10 ppmF groups (A/J *vs* 129P3/J mice), and 50 ppmF groups (A/J *vs* 129P3/J mice). Tables 1–3 show the proteins that were differentially expressed (p<0.05) in each comparison. Representative 2D map of each comparison is also shown in the Supplementary Information (Figures S1-S3). Quantitative intensity analysis showed 26 changed spots between control groups (Table 1). Among them, 14 spots were up-modulated, while were 12 down-regulated in control 129P3/J mice, when compared to control A/J mice. In general, the kidney proteome dataset was found to be significantly related with several metabolic and cellular processes pathways. Most of the 14 proteins up-modulated in the kidney of 129P3/J mice are related with metabolism (57.2%), while 28.6% are involved in cell processes and the remainder in information pathways (7.1%) and transport (7.1%). A similar pattern was observed for the proteins that were down-regulated in kidney 129P3/J mice. The respective percentages were 50.0, 25.0, 16.7 and 8.3 (Table 1). From the differentially expressed proteins in control groups, 10 were exclusively expressed in this comparison whereas 2, 6, and 8 proteins were also present in either 10 ppmF or 50 ppmF or both F-treated groups, respectively (Figure 1).

**Table 1:** Expression of differentially significant kidney proteins between control A/J *vs* control 129P3/J mice.

Spot n°.	Protein	aM_w_ (kDa)/p*I* Expt.	*^b^*Theor.	*^c^*Number of peptides/Score	*^d^*Difference(P value)	*^e^*Uniprot ID	*^f^*Biological Process
2	*Aconitate hydratase, mitochondrial*	91/4.71	82.5/7.4	6/99	↑129 (0.046)	Q99KI0	Metabolism
119/280	*ATP synthase subunit beta,* *mitochondrial*	33/5.155	51.8/5.0	16/1129	↑129(0.038)	P56480	Metabolism
151	*Pyruvate dehydrogenase E1 component* *subunit beta, mitochondrial*	36.5/5.1	35.8/5.4	8/434	↑129(0.011)	Q9D051	Metabolism
175	*Hydroxyacid oxidase 2*	38.5/7.94	38.7/7.6	11/529	↑129(0.012)	Q9NYQ2	Metabolism
217/221	*Medium-chain specific acyl-CoA* *dehydrogenase, mitochondrial*	42.5/8.055	43.6/7.69	15/715	↑129(0.001)	P45952	Metabolism
269	*Homogentisate 1,2-dioxygenase*	50/7.2	50/6.85	6/105	↑129(0.011)	O09173	Metabolism
384	*Sarcosine dehydrogenase, mitochondrial*	95/6.14	-	18/583	↑129(0.041)	Q99LB7	Metabolism
116	*Beta-lactamase-like protein 2*	32.5/8.885	32.8/5.9	14/198	↑129(0.043)	Q99KR3	Metabolism
7	*Serine/threonine-protein phosphatase* *PP1-alpha catalytic subunit*	38.5/5.675	37.4/5.9	9/122	↑129 (0.018)	P62137	Processes/Information pathways
317/321	*Catalase*	58/7.37	59.7/7.7	6/103	↑129(0.032)	P24270	Processes
396	*Aldehyde dehydrogenase family 1 member L1*	100/5.445	98.7/5.6	24/517	↑129(0.013)	Q8R0Y6	Processes
285	*Alpha-aminoadipic semialdehyde dehydrogenase*	52/6.22	55.9/6.0	13/374	↑129(0.001)	Q9DBF1	Processes
324/583	*60 kDa heat shock protein, mitochondrial*	59.5/4.875	58/5.35	22/992	↑129(0.022)	P63038	Information Pathways
413	*Coatomer subunit delta*	63/6.2	57.2/5.9	7/206	↑129(0.009)	Q5XJY5	Transport
3	*ATP synthase subunit delta, mitochondrial*	15.5/2.82	51.7/5.0	4/188	↓129(0.022)	P56480	Metabolism
171	*Alcohol dehydrogenase [NADP+]*	38/7.51	36.5/6.9	17/775	↓129(0.049)	Q9JII6	Metabolism
189	*Sorbitol dehydrogenase*	40.5/6.885	38.2/6.6	13/853	↓129(0.041)	Q64442	Metabolism
209	*Isovaleryl-CoA dehydrogenase, mitochondrial*	42.5/6.6	43/6.3	12/535	↓129(0.000)	Q9JHI5	Metabolism
233	*Ornithine aminotransferase, mitochondrial*	45/5.72	45.8/5.7	10/255	↓129(0.024)	P29758	Metabolism
237	*Creatine kinase U-type, mitochondrial*	45/8.05	43.2/7.3	9/133	↓129(0.033)	P30275	Metabolism
60	*Lactoylglutathione lyase*	27/4.48	20.7/5.25	37/365	↓129(0.001)	Q9CPU0	Processes
190	*Phosphotriesterase-related protein*	41/6.58	39.2/6.2	7/293	↓129(0.029)	Q60866	Processes
205	*40S ribosomal protein SA*	42.5/3.96	32.7/4.8	7/825	↓129(0.044)	P14206	Processes
99	*Proteasome subunit beta type-7*	30/5.885	25.3/5.8	7/85	↓129(0.041)	P70195	Information pathways
385/386/520	*Meprin A subunit* alpha	93/5.43	77.2/5.9	11/187	↓129(0.020)	P28825	Information pathways
355	*Serum albumin*	69.5/5.33	65.9/5.53	15/635	↓129(0.022)	P07724	Transport

a Experimental molecular weight (kDa)/p*I* of protein spot in the gel (Mean of min. and max.) based on the coordinates of landmark proteins. *^b^*Theoretical molecular weight (kDa)/p*I* of theoretical protein. *^c^*Number of peptides identified and score. *^d^*Differences in expression in relation to 129P3/J mice (↓ down-modulation; ↑ up-modulation); Individual *P* value after ANOVA. *^e^*Identification is based on protein ID from IPI (international protein index) protein database (http://www.uniprot.org/). *^f^*Category of protein based on its primary biological function according to Rison (2000) [18].

**Table 2:** Expression of differentially significant kidney proteins between 10 ppmF A/J *vs* 10 ppmF 129P3/J mice.

Spot n°.	Protein	aM_w_ (kDa)/p*I* Expt.	*^b^*Theor.	*^c^*Number of peptides/Score	*^d^*Difference (P value)		*^e^*Uniprot ID		*^f^*Biological Process
3	*ATP synthase subunit delta, mitochondrial*	15.5/2.82	51.7/5.0	4/188	↑129(0.040)	P56480		Metabolism	
182	*Aldose 1-epimerase*	40/6.625	37.8/6.3	4/146	↑129(0.016)	Q8K157		Metabolism	
210	*Aminoacylase-1*	42.5/5.94	45.8/5.9	18/831	↑129(0.013)	Q99JW2		Metabolism	
221/217	*Medium-chain specific acyl-CoA dehydrogenase, mitochondrial*	43/8.25	43.6/7.7	15/541	↑129(0.001)	P45952		Metabolism	
223	*Adenosylhomocysteinase*	42.5/6.6	43/6.3	15/559	↑129(0.047)	P50247		Metabolism	
261	*Fumarate hydratase, mitochondrial*	45/5.72	50/7.9	8/142	↑129(0.015)	P97807		Metabolism	
462	*Short/branched chain specific acyl-CoA dehydrogenase, mitochondrial*	42/6.11	44/6.0	2/66	↑129(0.047)	Q9DBL1		Metabolism	
467	*3-hydroxyanthranilate 3,4-dioxygenase*	34/6.415	32.8/6.1	8/181	↑129(0.039)	Q78JT3		Metabolism	
489	*Citrate synthase, mitochondrial*	43/9.1	49/8.2	3/107	↑129(0.035)	Q9CZU6		Metabolism	
142/146	*Aspartoacylase-2*	35.5/4.915	35.3/5.3	9/488	↑129(0.003)	Q91XE4		Processes	
7	*Serine/threonine-protein phosphatase PP1-alpha catalytic subunit*	38.5/5.675	37.4/5.9	9/122	↑129 (0.018)	P62137		Processes/Information pathways	
285	*Alpha-aminoadipic semialdehyde* dehydrogenase	52.5/6.34	55.9/6.0	13/374	↑129(0.005)	Q9DBF1		Processes	
317/321	*Catalase*	58/7.445	59.7/7.7	6/103	↑129(0.032)	P24270		Processes	
289	*Selenium-binding protein 1*	53.5/5.68	52.5/5.9	13/374	↑129(0.037)	P17563		Processes	
5	*Ester hydrolase C11orf54 homolog*	37/5.905	51.7/5.0	4/188	↓129(0.022)	Q91V76		Metabolism	
87	*Enoyl-CoA hydratase, mitochondrial*	28/8.615	28.5/7.78	13/353	↓129(0.017)	Q8BH95		Metabolism	
157	*L-lactate dehydrogenase B chain*	37/5.505	36.4/5.7	10/705	↓129(0.002)	P16125		Metabolism	
185	*3′(2′),5′-bisphosphate nucleotidase 1*	41/5.535	33.1/5.54	7/216	↓129(0.016)	Q9Z0S1		Metabolism	
209	*Isovaleryl-CoA dehydrogenase, mitochondrial*	43/6.715	43/6.3	12/535	↓129(0.021)	Q9JHI5		Metabolism	
233	*Ornithine aminotransferase, mitochondrial*	45/5.72	45.8/5.7	10/255	↓129(0.024)	P29758		Metabolism	
518	*Nucleoside diphosphate-linked moiety X motif 19, mitochondrial*	42/6.015	39.3/6.0	7/245	↓129(0.041)	P11930		Metabolism	
60	*Lactoylglutathione lyase*	27/4.48	20.7/5.25	37/365	↓129(0.001)	Q9CPU0		Processes	
85	*Rho GDP-dissociation inhibitor 1*	27/4.55	23.3/5.1		↓129(0.005)	Q99PT1		Processes	
512	*Na(+)/H(+) exchange regulatory cofactor NHE-RF3*	69.5/4.645	56.5/5.3	13/403	↓129(0.041)	Q9JIL4		Processes	
75	*GTP:AMP phosphotransferase, mitochondrial*	26.5/10.135	25.3/8.88	7/85	↓129(0.050)	Q9WTP7		Information pathways	
320	*Protein disulfide-isomerase A3*	59.5/5.73	54.3/5.7	16/362	↓129(0.043)	P27773		Information pathways	
385/386/520	*Meprin A subunit alpha*	89.5/5.5	77.2/5.9	11/187	↓129(0.002)	P28825		Information pathways	
400	*Heat shock protein 90, beta (Grp94), member 1*	99.5/3.99	92.5/4.74	24/515	↓129(0.011)	Q91V38		Information Pathways	
267	*Actin-related protein 3*	49.5/5.445	47.2/5.6	13/554	↓129(0.016)	Q99JY9		Structure	
311	*Vitamin D-binding protein*	57.5/4.76	51.9/5.2	9/109	↓129(0.028)	P21614		Transport	
355	*Serum albumin*	69.5/5.42	65.9/5.53	15/635	↓129(0.020)	P07724		Transport	

a Experimental molecular weight (kDa)/p*I* of protein spot in the gel (Mean of min. and max.) based on the coordinates of landmark proteins. *^b^*Theoretical molecular weight (kDa)/p*I* of theoretical protein. *^c^*Number of peptides identified and score. *^d^*Differences in expression in relation to 129P3/J mice (↓ down-modulation; ↑ up-modulation); individual *P* value after ANOVA. *^e^*Identification is based on protein ID from IPI (international protein index) protein database (http://www.uniprot.org/). *^f^*Category of protein based on its primary biological function according to Rison (2000) [18].

**Table 3:** Expression of differentially significant kidney proteins between control A/J *vs* control 129P3/J mice.

Spot n°.	Protein	aM_w_ (kDa)/p*I* Expt.	*^b^*Theor.	*^c^*Number of peptides/Score	*^d^*Difference (P value)	*^e^*Uniprot ID	*^f^*Biological Process
2	*Aconitate hydratase, mitochondrial*	90/4.77	82.5/7.4	6/99	↑129(0.029)	Q99KI0	Metabolism
35	*ATP synthase subunit d, mitochondrial*	24/5.16	18.6/5.53	8/402	↑129(0.036)	Q9DCX2	Metabolism
116	*Beta-lactamase-like protein 2*	32.5/6.04	32.7/5.9	7/198	↑129(0.045)	Q99KR3	Metabolism
133	*Hydroxyacyl-coenzyme A dehydrogenase, mitochondrial*	33.5/9.28	32995.07/8.3	8/255	↑129(0.035)	Q61425	Metabolism
175	*Hydroxyacid oxidase 2*	37/8.1	38.7/7.6	11/529	↑129(0.003)	Q9NYQ2	Metabolism
184	*Sorbitol dehydrogenase*	40.5/6.805	38.2/6.56	10/586	↑129(0.003)	Q64442	Metabolism
210	*Aminoacylase-1*	42.5/5.92	45.8/5.9	18/831	↑129(0.006)	Q99JW2	Metabolism
217/221	*Medium-chain specific acyl-CoA dehydrogenase, mitochondrial*	41.5/8.22	43.6/7.69	15/715	↑129(0.009)	P45952	Metabolism
269	*Homogentisate 1,2-dioxygenase*	49/7.41	50/6.85	6/105	↑129(0.002)	O09173	Metabolism
280/119	*ATP synthase subunit beta, mitochondrial*	51/4.345	51.7/5.0	16/1129	↑129(0.001)	P56480	Metabolism
360	*Propionyl-CoA carboxylase alpha chain, mitochondrial*	77/6.07	74.4/6.0	13/315	↑129(0.033)	Q91ZA3	Metabolism
378	*Acylamino-acid-releasing enzyme*	86/5.075	81.5/5.3	10/256	↑129(0.001)	Q8R146	Metabolism
491	*Argininosuccinate synthase*	44/9.41	46.6/8.4	14/321	↑129(0.001)	P16460	Metabolism
142/146	*Aspartoacylase-2*	36.5/4.91	35.3/5.3	9/488	↑129(0.003)	Q91XE4	Metabolism
285	*Alpha-aminoadipic semialdehyde dehydrogenase*	53/6.355	55.9/6.0	13/374	↑129(0.024)	Q9DBF1	Processes
321	*Catalase*	56/8.055	59.7/7.7	6/103	↑129(0.008)	P24270	Processes
298	*ATP synthase subunit alpha, mitochondrial*	51/8.62	55.3/8.28	15/552	↑129(0.026)	Q03265	Information Pathways
372	*Radixin*	84/6.305	68.5/5.91	9/125	↑129(0.015)	P26043	Structure
90	*Phosphoglycerate mutase 1*	28.5/7.235	28.7/6.75	23/324	↓129(0.003)	Q9DBJ1	Metabolism
157	*L-lactate dehydrogenase B chain*	37.5/5.49	36.4/5.7	10/705	↓129(0.009)	P16125	Metabolism
209	*Isovaleryl-CoA dehydrogenase, mitochondrial*	43/6.76	43/6.3	12/535	↓129(0.000)	Q9JHI5	Metabolism
233	*Ornithine aminotransferase, mitochondrial*	46/5.855	45.8/5.7	10/255	↓129(0.006)	P29758	Metabolism
244	*Glycine amidinotransferase, mitochondrial*	45/7.07	44.2/6.4	15/234	↓129(0.003)	Q9D964	Metabolism
518	*Nucleoside diphosphate-linked moiety X motif 19, mitochondrial*	41.5/5.99	39.3/6.0	7/245	↓129(0.018)	P11930	Metabolism
60	*Lactoylglutathione lyase*	27/4.525	20.7/5.25	10/365	↓129(0.003)	Q9CPU0	Processes
190	*Phosphotriesterase-related protein*	40.5/6.74	39.2/6.2	7/293	↓129(0.044)	Q60866	Processes
512	*Na(+)/H(+) exchange regulatory cofactor NHE-RF3*	70/4.665	56.5/5.3	13/403	↓129(0.009)	Q9JIL4	Processes
267	*Actin-related protein 3*	49.5/5.44	47.2/5.6	13/554	↓129(0.015)	Q99JY9	Structure
355	*Serum albumin*	70/5.42	65.9/5.53	15/635	↓129(0.026)	P07724	Transport
376	*Serotransferrin*	82/7.26	74.9/6.8	12/326	↓129(0.019)	Q921I1	Transport
385/386/520	*Meprin A subunit alpha*	96.5/5.545	77.2/5.9	11/187	↓129(0.014)	P28825	Information pathways

a Experimental molecular weight (kDa)/p*I* of protein spot in the gel (Mean of min. and max.) based on the coordinates of landmark proteins. *^b^*Theoretical molecular weight (kDa)/p*I* of theoretical protein. *^c^*Number of peptides identified and score. *^d^*Differences in expression in relation to 129P3/J mice (↓ down-modulation; ↑ up-modulation); individual *P* value after ANOVA. *^e^*Identification is based on protein ID from IPI (international protein index) protein database (http://www.uniprot.org/). *^f^*Category of protein based on its primary biological function according to Rison (2000) [18].

For the comparison between the A/J and 129P3/J mice treated with 10 ppmF, 14 proteins were increased and 17 diminished in kidney of 129P3/J. Among the increased proteins, 64.3% are related with metabolism, while 35.7% are associated with cell processes. Most of the decreased proteins are also related to metabolism (41.1%), followed by information pathways (23.6%), cell processes (17.6%), transport (11.8%) and structure (5.9%) (Table 2). From the differentially expressed proteins in 10 ppmF group, 15 were exclusively expressed in this comparison whereas 2, 6 and 8 proteins were also present in either control or 50 ppmF or in both groups, respectively (Figure 1).

Regarding the comparison between the groups treated with 50 ppmF, 18 proteins were significantly up-regulated and 13 down-modulated in kidney of 129P3/J mice when compared with A/J mice. Fourteen of eighteen enhanced proteins are associated with metabolism (77.8%), followed by processes (11.1%), information pathways (5.6%) and processes pathways (5.6%). Among the down-modulated proteins, most are also related to metabolism (46.2%), followed by cell processes (23.0%), transport (15.4%), information pathways (7.7%) and structure (7.7%) (Table 3). Among the differentially expressed proteins in kidney of animals treated with 50 ppmF, 11 proteins are exclusively expressed in this group while 6, 6 and 8 proteins are also present in either control or 10 ppmF or both groups, respectively (Figure 1).

Among the 8 proteins differentially expressed between the mice strains, regardless of the treatment with F, catalase, medium-chain specific acyl-CoA dehydrogenase and alpha-aminoadipic semialdehyde dehydrogenase were up-regulated, while isovaleryl-CoA dehydrogenase, ornithine aminotransferase, lactoylglutathione lyase, meprin A subunit alpha and albumin were down-regulated in the kidney of 129P3/J mice.

### Identification of Unique Proteins

A/J and 129P3/J mice exhibited 11 and 3 exclusive proteins, respectively. From these, 9 (64.3%) are related to metabolism, followed by cell processes (4 or 28.6%) and information pathways (1 or 7.1%). This profile was not altered upon exposure to F (Table 4).

**Table 4:** Expression of unique kidney proteins between A/J and 129P3/J mice.

Spot n°.	Protein	aM_w_ (kDa)/p*I* Expt.	*^b^*Theor.	*^c^*Number of peptides/Score	*^d^*Uniprot ID	*^e^*Biological Process
***Kidney A/J mice***						
563	*Transaldolase*	37.5/5.975	37.4/6.6	5/151	Q93092	Metabolism
564	*Isobutyryl-CoA dehydrogenase, mitochondrial*	40.5/6.905	42.8/7.2	6/202	Q9D7B6	Metabolism
565	*Short-chain specific acyl-CoA dehydrogenase, mitochondrial*	41/6.805	42.2/6.3	9/396	Q07417	Metabolism
567	*Cystathionine gamma-lyase*	43.5/7.99	43.6/7.6	5/163	Q8VCN5	Metabolism
568	*Hydroxymethylglutaryl-CoA synthase, cytoplasmic*	56/5.7	57.6/5.65	2/62	Q8JZK9	Metabolism
586	*Probable D-lactate dehydrogenase, mitochondrial*	37.5/8.23	19.1/6.2	2/84	Q7TNG8	Metabolism
562	*Thiomorpholine-carboxylate dehydrogenase*	37.5/5.09	33.5/5.44	8/395	O54983	Metabolism
560	*Phenazine biosynthesis-like domain-containing protein 2*	34/4.795	32/5.2	4/130	Q9CXN7	Process
561	*Biliverdin reductase A*	37.5/6.755	33.3/6.5	3/86	Q9CY64	Process
576	*Sorting nexin-5*	49.5/6.165	46.7/6.2	4/97	Q9D8U8	Process
558	*Serum amyloid P-component*	30/5.3	23.9/6.4	3/52	P12246	Information Pathways
***Kidney 129P3/J mice***						
534/535	*Peroxisomal acyl-coenzyme A oxidase 1*	20.5/9.99	74.6/8.6	7/227	Q9R0H0	Metabolism
552	*Nicotinate-nucleotide pyrophosphorylase [carboxylating]*	36/6.49	31.5/6.2	4/61	Q91X91	Metabolism
539	*Arsenite methyltransferase*	43/5.38	41.8/5.6	8/217	Q91WU5	Process

a Experimental molecular weight (kDa)/p*I* of protein spot in the gel (Mean of min. and max.) based on the coordinates of landmark proteins. *^b^*Theoretical molecular weight (kDa)/p*I* of theoretical protein. *^c^*Number of peptides identified and score. *^d^*Identification is based on protein ID from IPI (international protein index) protein database (http://www.uniprot.org/). *^e^*Category of protein based on its primary biological function according to Rison (2000) [18].

## Discussion

In the present study, we identified proteins potentially involved in renal F metabolism that are either exclusively or differentially expressed in A/J and 129P3/J mice. This highlights the molecular mechanisms underlying the differential metabolic handling of F by these two strains of mice. Exclusive proteins expressed in A/J or 129P3/J mice exhibited the same profile, regardless exposure to F. This suggests that the genetic background *per se* accounts for such differences between these two strains of mice. We have focused on identified proteins that may be associated with metabolic handling of F and water and renal functions. Unique metabolic proteins in kidney from A/J mice are involved in carbohydrate (probable D-lactate dehydrogenase), carbon (transaldolase), aminoacid (isobutyryl-CoA dehydrogenase, hydroxymethylglutaryl-CoA synthase (HMGCS2), cystathionine gamma-lyase (CSE), thiomorpholine-carboxylate dehydrogenase), and fatty acid [*short-chain specific acyl-CoA dehydrogenase* (SCAD)] metabolism. CSE is an enzyme that breaks down cystathione into cysteine and α-ketobutyrate and catalyses elimination of L-homoserine, L-cystine and L-cysteine producing ammonia and hydrogen sulfide (H_2_S) [19], [20]. Considering that the urine is the main excretion route for ingested F [1], the presence of CSE in A/J mice might contribute to increase the urinary pH, which can help to explain the higher urinary F excretion observed for this strain, when compared with the 129P3/J mice [10]. The pH-dependency found for urinary F excretion is due to the fact that F can cross cell membranes in general, including the walls of the renal tubules in the form of HF. Thus, the higher the urinary pH, the higher the concentration of F^−^ that remains in the tubule to be excreted in urine [12]. Recently, it was shown that the expression of HMGCS2 was increased fourfold in diabetic kidneys, which leads to increased renal ketogenesis and plays an important role in the pathogenesis of diabetic nephropathy in type 2 diabetes [21]. In our data, the presence of HMGCS2 in kidney of A/J mice might turn these animals more prone to nephropathy, which could impair F reabsorption in kidneys [10].

Besides presenting unique proteins involved in metabolism, A/J mice also expresses exclusive proteins involved in cell processes (phenazine biosynthesis-like domain containing protein 2 (PBLD), biliverdin reductase A (BVR) and sorting nexin-5) and information pathways [serum amyloid P-component (SAP)]. Among these, SAP constitutes amyloid deposits characterized by the ordered aggregation of normal globular proteins and peptides into insoluble fibrils, which disrupt tissue architecture and are associated with cell death [22]. The presence of SAP only in A/J mice might increase the probability of kidney damage that could account for their diminished capacity to reabsorb various solutes including F, helping to explain the higher urinary F excretion seen in this strain previously [10].

From those proteins found exclusively in kidneys of 129P3/J mice, the peroxisomal acyl-coenzymeA oxidase 1 (AOX), a fatty acid metabolic protein, is shown to be expressed in proximal tubules and enhancement of its activity is associated with the preservation of kidney function during ischemia [23]. Another exclusive protein called arsenite-methyltransferase, presented only in 129P3/J mice, is a detoxifying protein involved in the arsenic biotransformation and elimination in proximal tubule epithelial cells [24]. The presence of these proteins in 129P3/J but not in A/J mice suggest that the former might have a higher capacity to reduce renal damage caused by different hostile conditions, such as exposure to F. Thus, the 129P3/J mice would be able to maintain F reabsorption in kidneys even under exposure to high F doses [10].

As mentioned above, F exposure did not alter the profile of unique proteins in either strain of mice. However, among the proteins differentially expressed in the comparisons between the two strains, only 8 were present in the control, 10 and 50 ppmF groups (catalase, medium-chain specific acyl-CoA dehydrogenase and alpha-aminoadipic semialdehyde dehydrogenase (α-AASA), isovaleryl-CoA dehydrogenase, ornithine aminotransferase, lactoylglutathione lyase, meprin A subunit alpha and albumin). Some of these significantly altered proteins with potential roles to contribute for the intrinsic differences in F and water handling by A/J and 129P3/J mice are highlighted below. Meprin A, an information pathways related protein, is an enzyme that hydrolyzes protein and peptide substrates including components of the extracellular matrix [25]. It is highly expressed at the brush border membrane of proximal tubule cells of the kidney. Inbred strains of mice subjected to ischemia reperfusion that express low levels of meprin A in kidney have markedly less kidney damage [26]. Our data show that meprin A is consistently reduced in 129P3/J kidney in all experimental conditions. This suggests that this protein could act in concert with SAP to decrease renal damage caused by F in 129P3/J mice. Among the proteins related to cellular processes, it is important to highlight α-AASA dehydrogenase and catalase. α-AASA dehydrogenase metabolyzes irreversibly betaine aldehyde to betaine, which is the most effective osmoprotectant accumulated by eukariotic organisms to cope with osmotic stress [27]. This enzyme was increased in the 129P3/J kidney, regardless F exposure. This can explain the lower volume of water consistently ingested by the 129P3/J mice throughout the study, which led us to adjust water F concentrations throughout the experiment in order that both strains had the same amount of F intake from the water [10]. The increased expression of the antioxidant enzyme catalase might indicate a higher capacity of the 129P3/J mice to deal with oxidative stress [28].

Two and 6 proteins with differential expression between the two strains in the control group were also identified upon exposure to 10 and 50 ppmF, respectively. Low F level increased the expression of serine/threonine-protein phosphatase PP1 and ATP synthase subunit delta. High F level kidney up-expressed aconitate hydratase, ATP synthase subunit beta, hydroxyacid oxidase 2, homogentisate 1,2-dioxygenase and beta-lactamase-like protein 2 and down-expressed phosphotriesterase-related protein. Besides, 6 proteins presented altered expression only in F-treated groups. Aminoacylase-1 and aspartoacylase-2 were increased, whereas L-lactate dehydrogenase B chain, nucleoside diphosphate-linked moiety X motif 19, Na(+)/H(+) exchange regulatory cofactor NHE-RF3 (PDZK1) and actin-related protein 3 were diminished in 129P3/J kidney. These proteins may act as molecular targets for the differential F metabolism between these strains induced by the treatment. Protein phosphatase 1 (PP1) is a serine/threonine protein phosphatase involved in diverse cellular processes, such as transcription, replication, pre-mRNA splicing, protein synthesis, carbohydrate metabolism, neuronal signaling, cell survival, and cell cycle progression [29], [30]. Phosphatases typically function antagonistically with kinases to achieve fine control over the phosphorylation state of proteins. Phosphatases are widely expressed enzymes that mediate the functional regulation of many proteins, including some renal channels and transporters such as the inwardly rectifying K^+^ channel, Na^+^-K^+^-Cl^−^ cotransporter (NKCC1), CFTR, epithelial Na^+^ channel (ENaC), aquaporin-2 (AQP2) and Na^+^/H^+^ exchanger 3 (NHE3) [30], [31], [32], [33], [34], [35], [36]. In general, these ions and water channels are responsible to maintain the urine normal volume and acid-base status under varying physiological conditions and are under direct or indirect phosphorylation state control [37], [38]. It was shown that the prevention of phosphorylation of specific sites in AQP2 increases localization of AQP2 vesicles to the apical plasma membrane leading to water reabsorption and urine concentration [38]. Thus, we could speculate that the fact that 129P3/J mice excrete less urine could be possibly explained by the PP1-mediated enhancement of AQP2 vesicles trafficking, which should be confirmed in future studies.

PDZK1 is a scaffold protein that connects plasma membrane proteins and regulatory components, regulating their surface expression in epithelial cells apical domains. Within the kidney, PDZK1 is localized exclusively in the brush border of the proximal tubule and interacts with several renal proteins including NHE3, a Na-H exchanger, and CFEX, a Cl-anion exchanger [39]. These exchanger transporters play principal roles in the reabsorption of Na^+^ and Cl^−^ in the proximal tubule of the mammalian kidney. Besides regulating reabsorption of filtered solutes, PDZK1 also plays a direct and essential role in maintaining normal brush border expression and function of CFEX in the proximal tubule *in vivo* [39]. The diminished expression of PDZK1 in kidney of 129P3/J mice may indicate an undisclosed impaired ability of ion reabsorption by this strain, which is consistent with the lower volume of urine excreted by these mice.

We conclude that the renal proteome indicates several specific target proteins, both strain and F-induced, which possibly regulate the water and F metabolism in kidney of mice with distinct susceptibilities to F. In addition, although we did not focus in the correlation between target kidney proteins and DF, we found that some of those changed proteins are also codified by chromosomes 2 (13 proteins: sarcosine dehydrogenase, catalase, sorbitol dehydrogenase, isovaleryl-CoA dehydrogenase, creatine kinase U-type, phosphotriesterase-related protein, proteasome subunit beta type-7, adenoxylhomocysteinase, protein disulfide-isomerase A3, argininosuccinate synthase, glycine amidinotransferase, biliverdin reductase A and sorting nexin-5) and 11 (3 proteins: peroxisomal acyl-coenzyme A oxidase 1, ATP synthase subunit d and Rho GDP-dissociation inhibitor 1), previously characterized to determine susceptibility and resistance to DF in A/J and 129P3/J mice, respectively [40], [41]. This correlation may provide a database for future hypothesis-driven researches.

## Supporting Information

Figure S1 **2D gel analysis of renal proteome.** Representative 2D maps of control kidneys. Selected spots in green represent those with differential expression in the comparison between control A/J (A) *vs* control 129P3/J mice (B). In Figure B, spot identification numbers in boundaries or not represents increases or decreases in protein expression when compared to A/J, respectively (Figure A). Dashed lines represent unique spots in the AJ group (A) and 129P3/J group (B), regardless exposure to F.
(TIF)

Figure S2 **2D gel analysis of renal proteome.** Representative 2D maps of 10 ppmF treated-groups. Selected spots in green represent those with differential expression in the comparison between 10 ppmF treated- A/J (A) *vs* 10 ppmF treated- 129P3/J mice (B). In Figure B, spot identification numbers in boundaries or not represents increases or decreases in protein expression when compared to A/J, respectively (Figure A).
(TIF)

Figure S3 **2D gel analysis of renal proteome.** Representative 2D maps of 50 ppmF treated-groups. Selected spots in green represent those with differential expression in the comparison between 50 ppmF treated- A/J (A) *vs* 50 ppmF treated- 129P3/J mice (B). In Figure B, spot identification numbers in boundaries or not represents increases or decreases in protein expression when compared to A/J, respectively (Figure A).
(TIF)

Figure S4 **2D gel variability analysis.** Scatter plot of binary comparisons among the ratios of relative spots volumes detected in the representative gel (replicate 1) and the respective replicates (replicates 2 and 3). (A) Control A/J mice. (B) 10 ppmF treated-A/J mice. (C) 50 ppmF treated-A/J. (D) Control 129P3/J mice. (E) 10 ppmF treated-129P3/J mice. (F) 50 ppmF treated-129P3/J.
(TIF)

## References

[1] Buzalaf MA, Levy SM (2011) Fluoride intake of children: considerations for dental caries and dental fluorosis. In: Lussi, A.; Huysmans, M.C.D.N.J.M.; Weber, H.P, editors. Fluoride and the oral enviroment. Monographs in Oral Science. Basel: Karger. 1–19. 10.1159/000325101 21701188

[2] Khan A, Moola MH, Cleaton-Jones P (2005) Global trends in dental fluorosis from 1980 to 2000: a systematic review. SADJ 60: 418–421. 16438356

[3] Beltran-Aguilar ED, Barker LK, Canto MT, Dye BA, Gooch BF, et al (2005) Surveillance for dental caries, dental sealants, tooth retention, edentulism, and enamel fluorosis–United States, 1988–1994 and 1999–2002. MMWR Surveill Summ 54: 1–43. 16121123

[4] Bronckers AL, Lyaruu DM, DenBesten PK (2009) The impact of fluoride on ameloblasts and the mechanisms of enamel fluorosis. J Dent Res 88: 877–893. 19783795 10.1177/0022034509343280 PMC3318083

[5] Everett ET (2011) Fluoride's effects on the formation of teeth and bones, and the influence of genetics. J Dent Res 90: 552–560. 20929720 10.1177/0022034510384626 PMC3144112

[6] Huang H, Ba Y, Cui L, Cheng X, Zhu J, et al (2008) COL1A2 gene polymorphisms (Pvu II and Rsa I), serum calciotropic hormone levels, and dental fluorosis. Community Dent Oral Epidemiol 36: 517–522. 18284430 10.1111/j.1600-0528.2007.00424.x

[7] Everett ET, McHenry MA, Reynolds N, Eggertsson H, Sullivan J, et al (2002) Dental fluorosis: variability among different inbred mouse strains. J Dent Res 81: 794–798. 12407097 10.1177/0810794

[8] Mousny M, Banse X, Wise L, Everett ET, Hancock R, et al (2006) The genetic influence on bone susceptibility to fluoride. Bone 39: 1283–1289. 16920415 10.1016/j.bone.2006.06.006

[9] Mousny M, Omelon S, Wise L, Everett ET, Dumitriu M, et al (2008) Fluoride effects on bone formation and mineralization are influenced by genetics. Bone 43: 1067–1074. 18755305 10.1016/j.bone.2008.07.248 PMC2664744

[10] Carvalho JG, Leite AL, Yan D, Everett ET, Whitford GM, et al (2009) Influence of genetic background on fluoride metabolism in mice. J Dent Res 88: 1054–1058. 19828896 10.1177/0022034509347249 PMC3318017

[11] Buzalaf MAR, Whitford GM (2011) Fluoride metabolism. In: Lussi, A.; Huysmans, M.C.D.N.J.M.; Weber, H.P, editors. Fluoride and the oral environment Monographs in Oral Science. Basel: Karger. 20–36. 10.1159/000325107 21701189

[12] Whitford GM, Pashley DH, Stringer GI (1976) Fluoride renal clearance: a pH-dependent event. Am J Physiol 230: 527–532. 1259032 10.1152/ajplegacy.1976.230.2.527

[13] Schiffl HH, Binswanger U (1980) Human urinary fluoride excretion as influenced by renal functional impairment. Nephron 26: 69–72. 7412962 10.1159/000181954

[14] Taves DR (1968) Determination of submicromolar concentrations of fluoride in biological samples. Talanta 15: 1015–1023. 18960400 10.1016/0039-9140(68)80109-2

[15] Whitford GM (1996) The metabolism and toxicity of fluoride. Monogr Oral Sci 16 Rev 2: 1–153. 8813212

[16] Tilton RG, Haidacher SJ, Lejeune WS, Zhang X, Zhao Y, et al (2007) Diabetes-induced changes in the renal cortical proteome assessed with two-dimensional gel electrophoresis and mass spectrometry. Proteomics 7: 1729–1742. 17436268 10.1002/pmic.200700017

[17] Candiano G, Bruschi M, Musante L, Santucci L, Ghiggeri GM, et al (2004) Blue silver: a very sensitive colloidal Coomassie G-250 staining for proteome analysis. Electrophoresis 25: 1327–1333. 15174055 10.1002/elps.200305844

[18] Rison SC, Hodgman TC, Thornton JM (2000) Comparison of functional annotation schemes for genomes. Funct Integr Genomics 1: 56–69. 11793222 10.1007/s101420000005

[19] Levonen AL, Lapatto R, Saksela M, Raivio KO (2000) Human cystathionine gamma-lyase: developmental and in vitro expression of two isoforms. Biochem J 347 Pt 1: 291–295. PMC1220959 10727430

[20] Yang G, Wu L, Bryan S, Khaper N, Mani S, et al (2010) Cystathionine gamma-lyase deficiency and overproliferation of smooth muscle cells. Cardiovasc Res 86: 487–495. 20051385 10.1093/cvr/cvp420

[21] Zhang D, Yang H, Kong X, Wang K, Mao X, et al (2011) Proteomics analysis reveals diabetic kidney as a ketogenic organ in type 2 diabetes. Am J Physiol Endocrinol Metab 300: E287–295. 20959534 10.1152/ajpendo.00308.2010

[22] Gillmore JD, Hawkins PN (1999) Amyloidosis and the respiratory tract. Thorax 54: 444–451. 10212113 10.1136/thx.54.5.444 PMC1763786

[23] Portilla D, Dai G, Peters JM, Gonzalez FJ, Crew MD, et al (2000) Etomoxir-induced PPARalpha-modulated enzymes protect during acute renal failure. Am J Physiol Renal Physiol 278: F667–675. 10751229 10.1152/ajprenal.2000.278.4.F667

[24] Peraza MA, Carter DE, Gandolfi AJ (2003) Toxicity and metabolism of subcytotoxic inorganic arsenic in human renal proximal tubule epithelial cells (HK-2). Cell Biol Toxicol 19: 253–264. 14686617 10.1023/b:cbto.0000003970.60896.49

[25] Kronenberg D, Bruns BC, Moali C, Vadon-Le Goff S, Sterchi EE, et al (2010) Processing of procollagen III by meprins: new players in extracellular matrix assembly? J Invest Dermatol 130: 2727–2735. 20631730 10.1038/jid.2010.202

[26] Bond JS, Matters GL, Banerjee S, Dusheck RE (2005) Meprin metalloprotease expression and regulation in kidney, intestine, urinary tract infections and cancer. FEBS Lett 579: 3317–3322. 15943977 10.1016/j.febslet.2005.03.045

[27] Roberts MF (2000) Osmoadaptation and osmoregulation in archaea. Front Biosci 5: D796–812. 10966877 10.2741/roberts

[28] Blaszczyk I, Grucka-Mamczar E, Kasperczyk S, Birkner E (2010) Influence of methionine upon the activity of antioxidative enzymes in the kidney of rats exposed to sodium fluoride. Biol Trace Elem Res 133: 60–70. 19488683 10.1007/s12011-009-8412-z

[29] Aggen JB, Nairn AC, Chamberlin R (2000) Regulation of protein phosphatase-1. Chem Biol 7: R13–23. 10662690 10.1016/s1074-5521(00)00069-7

[30] Ceulemans H, Bollen M (2004) Functional diversity of protein phosphatase-1, a cellular economizer and reset button. Physiol Rev 84: 1–39. 14715909 10.1152/physrev.00013.2003

[31] Becchetti A, Malik B, Yue G, Duchatelle P, Al-Khalili O, et al (2002) Phosphatase inhibitors increase the open probability of ENaC in A6 cells. Am J Physiol Renal Physiol 283: F1030–1045. 12372779 10.1152/ajprenal.00011.2002

[32] Darman RB, Flemmer A, Forbush B (2001) Modulation of ion transport by direct targeting of protein phosphatase type 1 to the Na-K-Cl cotransporter. J Biol Chem 276: 34359–34362. 11466303 10.1074/jbc.C100368200

[33] Li D, Aperia A, Celsi G, da Cruz e Silva EF, Greengard P, et al (1995) Protein phosphatase-1 in the kidney: evidence for a role in the regulation of medullary Na(+)-K(+)-ATPase. Am J Physiol 269: F673–680. 7503233 10.1152/ajprenal.1995.269.5.F673

[34] Mori Y, Kawasaki A, Takamaki A, Kitano I, Yoshida R, et al (2001) Ca(2+)-dependent inhibition of inwardly rectifying K(+) channel in opossum kidney cells. Jpn J Physiol 51: 371–380. 11492962 10.2170/jjphysiol.51.371

[35] Valenti G, Procino G, Carmosino M, Frigeri A, Mannucci R, et al (2000) The phosphatase inhibitor okadaic acid induces AQP2 translocation independently from AQP2 phosphorylation in renal collecting duct cells. J Cell Sci 113 (Pt 11): 1985–1992. 10.1242/jcs.113.11.1985 10806109

[36] Dynia DW, Steinmetz AG, Kocinsky HS (2010) NHE3 function and phosphorylation are regulated by a calyculin A-sensitive phosphatase. Am J Physiol Renal Physiol 298: F745–753. 20015946 10.1152/ajprenal.00182.2009 PMC2838583

[37] Moeller HB, Knepper MA, Fenton RA (2009) Serine 269 phosphorylated aquaporin-2 is targeted to the apical membrane of collecting duct principal cells. Kidney Int 75: 295–303. 18843259 10.1038/ki.2008.505 PMC3502047

[38] Moeller HB, Praetorius J, Rutzler MR, Fenton RA (2010) Phosphorylation of aquaporin-2 regulates its endocytosis and protein-protein interactions. Proc Natl Acad Sci U S A 107: 424–429. 19966308 10.1073/pnas.0910683107 PMC2806726

[39] Thomson RB, Wang T, Thomson BR, Tarrats L, Girardi A, et al (2005) Role of PDZK1 in membrane expression of renal brush border ion exchangers. Proc Natl Acad Sci U S A 102: 13331–13336. 16141316 10.1073/pnas.0506578102 PMC1201624

[40] Everett ET, Yan D, Weaver M, Liu L, Foroud T, et al (2009) Detection of dental fluorosis-associated quantitative trait Loci on mouse chromosomes 2 and 11. Cells Tissues Organs 189: 212–218. 18701810 10.1159/000151383 PMC2669892

[41] Everett ET, Yin Z, Yan D, Zou F (2011) Fine mapping of dental fluorosis quantitative trait loci in mice. Eur J Oral Sci 119 Suppl 18–12. 22243220 10.1111/j.1600-0722.2011.00868.x PMC3402502

