# Supplementary figures and images for: Renal Proteome in Mice with Different Susceptibilities to Fluorosis
Source: PLoS One. 2013 Jan 4;8(1):e53261. doi: 10.1371/journal.pone.0053261 (PMC3537663; doi:10.1371/journal.pone.0053261)

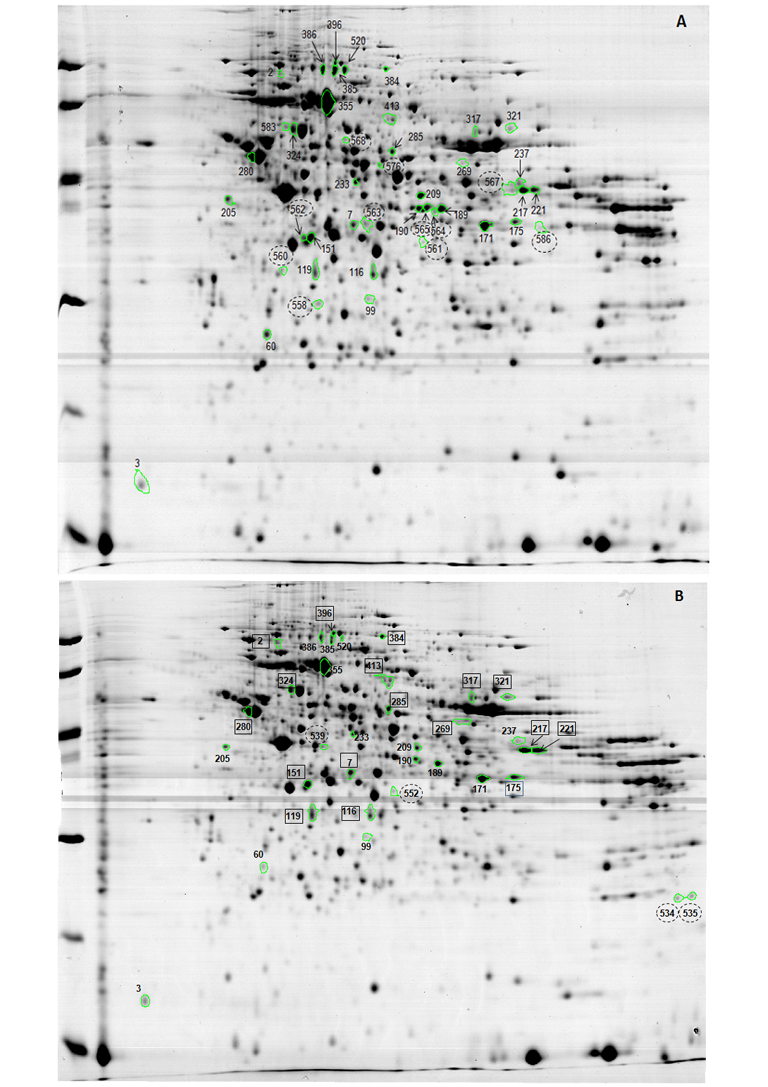

Supplement: Figure S1 — 2D gel analysis of renal proteome. Representative 2D maps of control kidneys. Selected spots in green represent those with differential expression in the comparison between control A/J (A) vs control 129P3/J mice (B). In Figure B, spot identification numbers in boundaries or not represents increases or decreases in protein expression when compared to A/J, respectively (Figure A). Dashed lines represent unique spots in the AJ group (A) and 129P3/J group (B), regardless exposure to F. (TIF) [file pone.0053261.s001.tif]

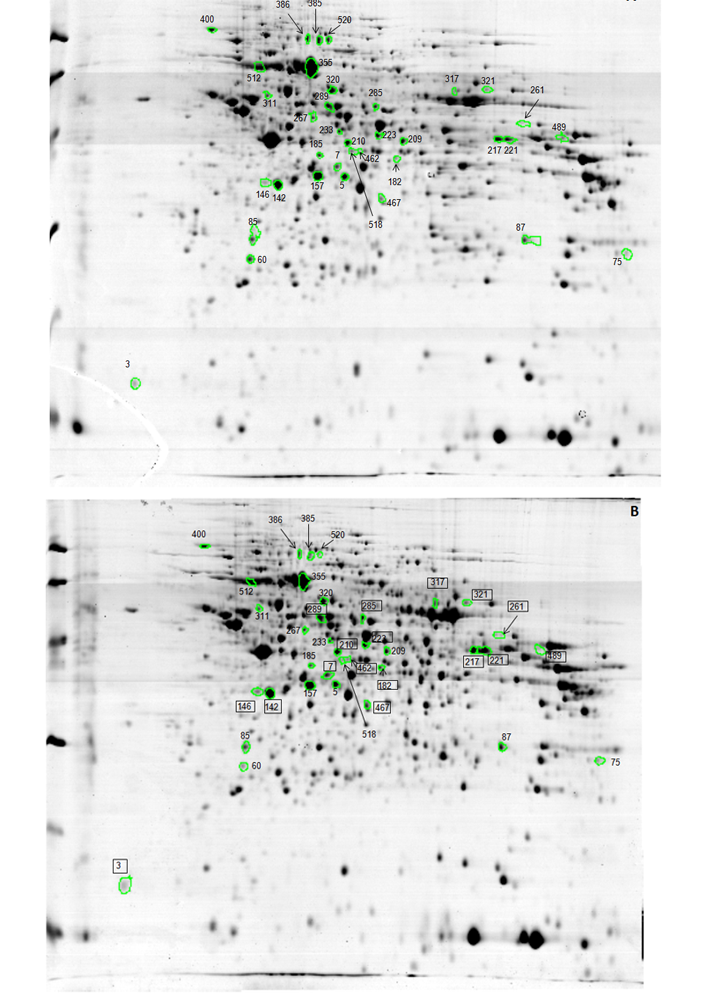

Supplement: Figure S2 — 2D gel analysis of renal proteome. Representative 2D maps of 10 ppmF treated-groups. Selected spots in green represent those with differential expression in the comparison between 10 ppmF treated- A/J (A) vs 10 ppmF treated- 129P3/J mice (B). In Figure B, spot identification numbers in boundaries or not represents increases or decreases in protein expression when compared to A/J, respectively (Figure A). (TIF) [file pone.0053261.s002.tif]

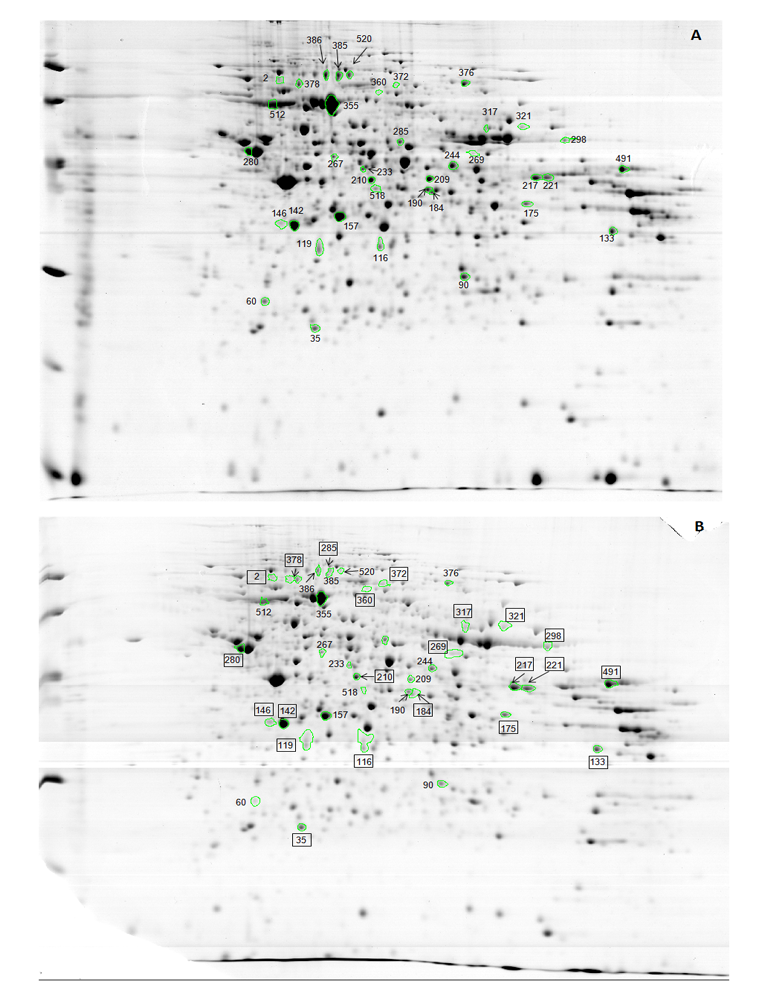

Supplement: Figure S3 — 2D gel analysis of renal proteome. Representative 2D maps of 50 ppmF treated-groups. Selected spots in green represent those with differential expression in the comparison between 50 ppmF treated- A/J (A) vs 50 ppmF treated- 129P3/J mice (B). In Figure B, spot identification numbers in boundaries or not represents increases or decreases in protein expression when compared to A/J, respectively (Figure A). (TIF) [file pone.0053261.s003.tif]

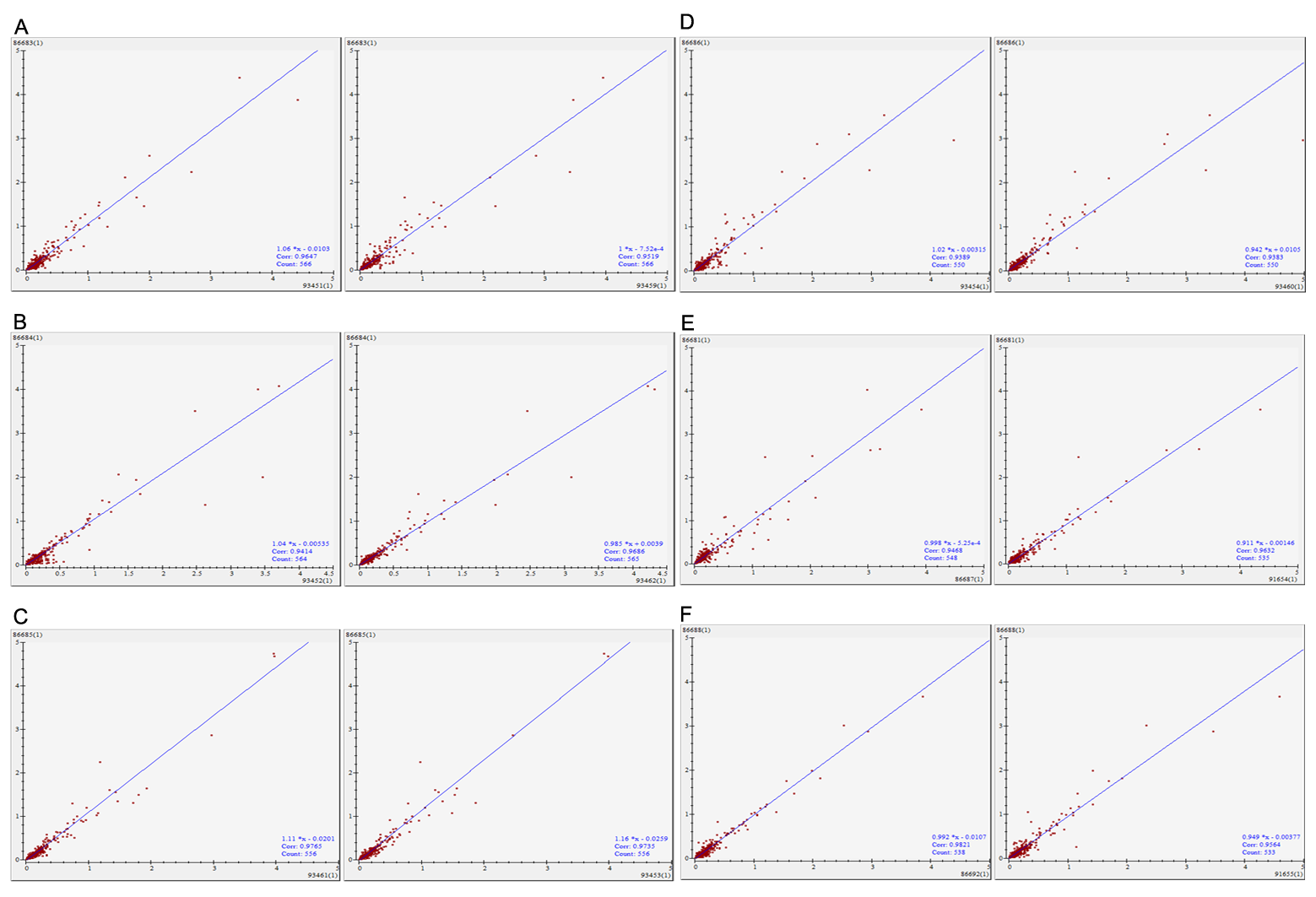

Supplement: Figure S4 — 2D gel variability analysis. Scatter plot of binary comparisons among the ratios of relative spots volumes detected in the representative gel (replicate 1) and the respective replicates (replicates 2 and 3). (A) Control A/J mice. (B) 10 ppmF treated-A/J mice. (C) 50 ppmF treated-A/J. (D) Control 129P3/J mice. (E) 10 ppmF treated-129P3/J mice. (F) 50 ppmF treated-129P3/J. (TIF) [file pone.0053261.s004.tif]
